# Acute Nicotine Poisoning From Nicotine Pouch Use in Adolescents: A Case Report of Two Pediatric Cases

**DOI:** 10.7759/cureus.103698

**Published:** 2026-02-16

**Authors:** Vasiliki Gketsi, Amvrosios Orfanidis, Aikaterini Gkrepi, Margarita Efthalia Ε Papasavva, Dimitra Florou, Ilektra Kyrochristou, Apostolia Balta, Vassiliki A Boumba

**Affiliations:** 1 Department of Pediatrics, General Hospital of Ioannina "G. Hatzikosta", Ioannina, GRC; 2 Department of Forensic Medicine and Toxicology, Faculty of Medicine, School of Health Sciences, University of Ioannina, Ioannina, GRC; 3 Faculty of Medicine, School of Health Sciences, University of Ioannina, Ioannina, GRC

**Keywords:** acute nicotine intoxication, adolescents, children, nicotine pouches, snuz

## Abstract

Acute nicotine poisoning is a well-studied clinical condition in both adults and children. However, poisoning resulting from the absorption of nicotine through the oral mucosa, as with modern nicotine pouches, is a complex and emerging field of medical research. This process is influenced by various factors that require further investigation, especially in the vulnerable age group of minors under 18 years, for whom the use of these products is legally prohibited. Based on two incidents of acute nicotine poisoning following the use of nicotine pouches in pediatric patients, the clinical and toxicological aspects of such conditions are discussed. The incidents concerned a 15-year-old boy and a 13-year-old girl. Both patients experienced symptoms consistent with nicotine poisoning, such as dizziness, nausea, vomiting, and loss of consciousness, which appeared within 90 and 20 minutes, respectively, after using the products. Both patients improved with supportive care. Samples taken upon their admission for toxicology testing confirmed acute nicotine poisoning with serum nicotine levels of 134 ng/ml and 266 ng/ml, respectively. Nicotine pouches, while promoted as a reduced-harm alternative to tobacco products for adult smokers, are not risk-free products. The distinction between "reduced harm" and "risk-free" is crucial and must be a central point in the discussion on legislative regulation and public health.

## Introduction

Nicotine is a potent toxic alkaloid that can cause acute poisoning with a wide range of clinical symptoms. Its action primarily lies as a neuroregulatory agent on neuronal nicotinic acetylcholine receptors in both the central and peripheral nervous systems, stimulating dopamine release via the mesolimbic pathway [1]. Toxicity is traditionally considered dose-dependent and biphasic. A stimulatory phase appears 15 minutes to one hour after intake, followed by a depressive phase lasting four to six hours, depending on the amount consumed [2]. The usual early symptoms include hypersalivation, bronchorrhea, and emesis, which may be accompanied by hyperpnea, hypertension, and tachycardia. Seizures, tremors, and muscle fasciculations have also been reported in the initial phase [3]. The depressive phase includes bradycardia, hypotension, and, most notably, neuromuscular blockade, leading to weakness, hypoventilation, apnea, and ultimately, cardiovascular collapse [3]. The lethal dose of nicotine for adults is traditionally reported to be between 30 and 60 mg, or 0.8 to 1.0 mg per kg of body weight, with a nicotine half-life of two to three hours [4,5]. Numerous cases of accidental or intentional poisoning have been recorded in the literature, some of which resulted in death, including incidents related to products characterized as "safe, alternative nicotine solutions" [6,7].

Nicotine can be absorbed by the body through various routes. These include the alveolar surface of the lungs (from smoked tobacco products such as cigarettes, e-cigarettes, and hookahs), the intestinal mucosa (from ingestion of tobacco or nicotine-containing substances, such as tablets, candies, and gummies), the skin (via nicotine patches), and the oral mucosa (via products such as gums, lozenges, chewing tobacco, and nicotine pouches/snus). Absorption through the oral mucosa presents specific peculiarities. Nicotine is a weak base, and its absorption is much more effective when it is in its free, non-ionized form. In an acidic environment, nicotine ionizes, which significantly reduces its absorption. To improve nicotine absorption, nicotine pouches typically contain buffering agents that increase the pH of the oral cavity. The speed and extent of absorption are determined by the product's pH [8,9], the total nicotine content of the pouch [10], the time it remains in the mouth, and the frequency of use. Although absorption is slower compared to smoking and vaping [11,12], as nicotine is not directly inhaled into the lungs, this route bypasses first-pass metabolism in the liver. This results in nicotine entering the bloodstream more directly and remaining in circulation for a longer period of time.

The increasing abuse of various commercial oral nicotine products -- in the form of pouches, lozenges, tablets, and gummies -- has gained exceptionally high popularity among adolescents in recent years, making them a new trend for this age group [13, 14]. Flavored nicotine products are considered by adolescents to be more attractive, less harmful, and less addictive compared to unflavored ones [15]. A significant aspect of this epidemic is that the use of flavored products is more widespread among adolescents (12-17 years: 80%) and young adults (18-24 years: 73%) compared to older smokers (≥65 years: 29%) [16]. For many adolescents, these products are their first type of tobacco. Furthermore, tobacco companies and related product manufacturers have been reported to target especially young people, adolescents, and even children, by adding flavoring chemicals and flavors that cover the harsh taste of tobacco and reduce local mucosal irritation during use [15]. This approach constitutes a potential link between marketing strategies and public health outcomes. The combination of attractive flavors, vivid colors, and easy accessibility (despite the ban on sales to minors) creates conditions where the "reduced harm" information intended for adult smokers is misinterpreted by adolescents as "risk-free." 

"Modern" oral nicotine pouches are a nicotine product similar to Swedish snus. Nicotine pouches are now the second most frequently used tobacco product by children and adolescents, after e-cigarettes [15]. They belong to the category of smokeless tobacco products and are placed between the upper lip and the gums. They contain nicotine, sweeteners, plant fibers, water, pH regulators, and flavorings. Although companies advertise that their processing keeps nitrosamines, heavy metals, and other potentially harmful chemical compounds at low levels, the risk of adverse effects is not negligible. Some types contain nicotine quantities similar to or even higher (up to five times more) than those in cigarettes. Their use has been associated with all the known acute and chronic harmful effects of nicotine, such as gastrointestinal disorders, allergic reactions, fainting episodes, dental diseases, cardiovascular diseases, and addiction.

## Case presentation

In both cases, a 15-year-old boy and a 13-year-old girl were involved in the region of Epirus, in mainland Greece, in 2023. The respective hospitalizations took place at a local hospital. Blood and urine samples for toxicological and other laboratory analyses were collected upon admission. Systematic toxicological analysis was performed with a standard analytical method routinely applied for the purpose. Ethanol and volatiles analysis was performed in whole blood by headspace gas chromatography-flame ionization detection (HS-GC-FID) [17,18]. Screening for opiates, benzodiazepines, amphetamines, cannabinoids, and cocaine metabolites in urine and blood was performed by immunoassays (SYVA, Abbott Park, IL). Toxicological screening for the presence of common drugs and poisons was carried out by gas chromatography-mass spectrometry (GC-MS) technique in full-scan mode on blood and urine extracts following solid-phase extraction. Nicotine levels in blood were determined with the routine procedure used for screening and quantification purposes [19].

Case A: 15-year-old boy

**Figure 1:**
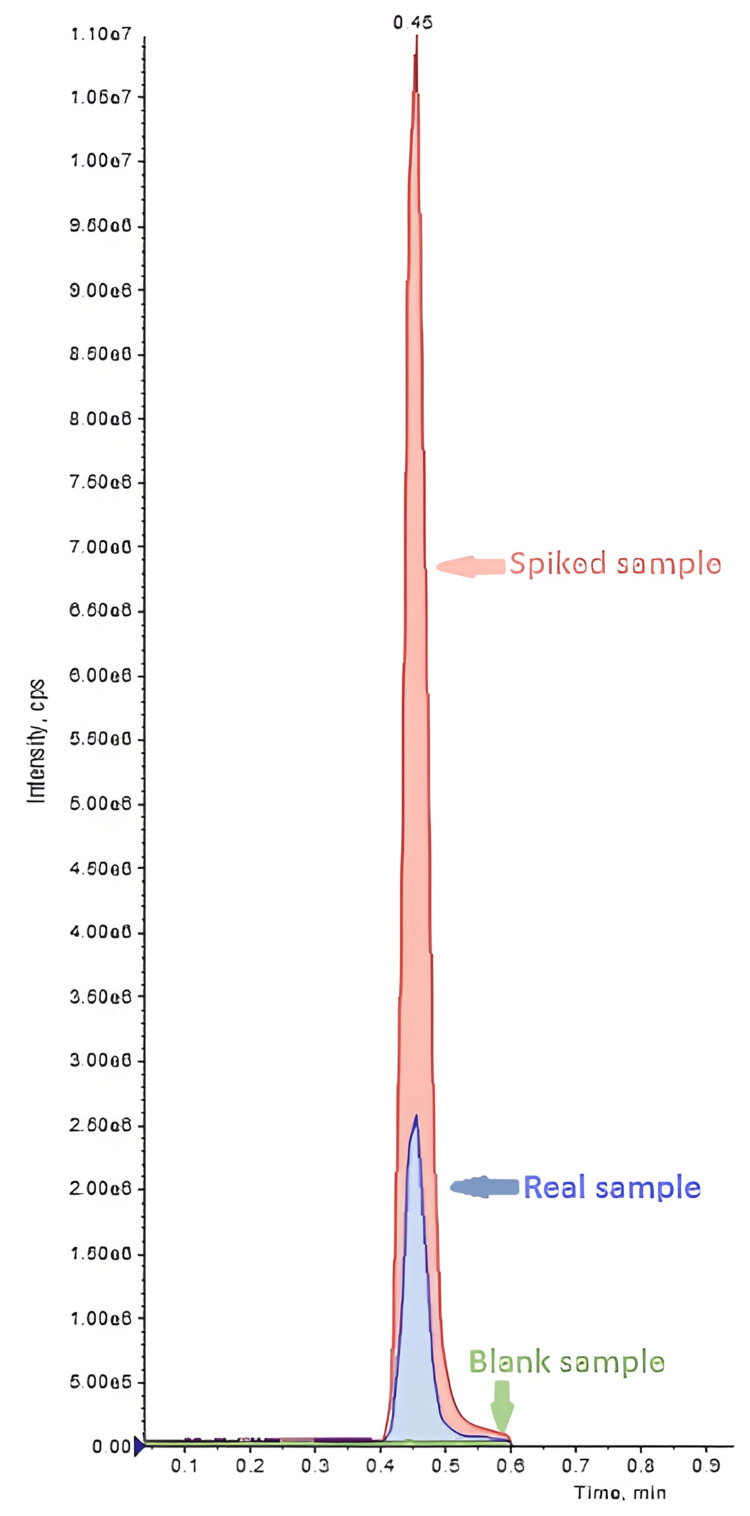
Chromatographic peaks (liquid chromatography-mass spectrometry) of a nicotine-spiked blood sample at a concentration of 450 ng/mL, a blank blood sample, and a real sample at a concentration of 134 ng/mL (case A).

The patient presented to the hospital with dizziness, nausea, vomiting, diarrhea, and tremor. He reported that about 90 minutes before his admission to the Emergency Department, he had used nicotine pouches with a friend who experienced similar symptoms. Upon admission, his initial vital signs showed a temperature of 36.5^∘^C, a heart rate of 100 beats per minute, a respiratory rate of 15 per minute, blood pressure of 124/81 mmHg, and oxygen saturation of 98% on room air. His body weight was 70 kg, height 180.5 cm, and body mass index was 21.49 kg/m^2^. The physical examination was otherwise normal, as were fundoscopy, electrocardiogram, and laboratory tests. The patient reported occasional alcohol and coffee consumption, as well as frequent consumption of energy drinks (about twice a week), but not on the day of admission. Activated charcoal was administered on admission within the recommended therapeutic window of one to two hours [20]. After a few hours, the symptoms subsided, and his blood pressure and pulse returned to normal (Table 1). Blood and urine samples collected upon admission were toxicologically analyzed. The results were negative for opioids, cannabinoids, benzodiazepines, amphetamines, coca alkaloids, and ethanol. The nicotine levels in the patient's blood and urine were indicative of acute poisoning, as shown in Figure 1 and Table 2.

Case B: 13-year-old girl

The patient presented to the hospital with dizziness, episodes of vertigo, headache, and a brief loss of consciousness lasting about five minutes, immediately prior to her admission. She reported using nicotine pouches 20 minutes before the onset of symptoms. Upon arrival, her initial vital signs showed a temperature of 36.7^∘^C, a heart rate of 105 beats per minute, a respiratory rate of 15 per minute, blood pressure of 134/73 mmHg, and oxygen saturation of 100% on room air. Her body weight was 47.5 kg. The physical examination was otherwise normal, as were fundoscopy, electrocardiogram, and laboratory tests. She also reported occasional smoking and use of nicotine pouches, as well as frequent consumption of energy drinks over the past year. After receiving intravenous hydration, her condition improved within a few hours. Her blood pressure and pulse also returned to normal (Table 1). Blood and urine samples collected upon admission were toxicologically analyzed, with the results being negative for opioids, cannabinoids, benzodiazepines, amphetamines, coca alkaloids, and ethanol. Her blood and urine nicotine levels were indicative of acute poisoning (Table 2).

**Table 1:** Changes in patients' vital signs. Heart rate normal range for children >10 years old (per minute): 60-100; blood pressure normal range for adolescents (mmHg): systolic blood pressure: 110-127 (females)/113-131 (males); diastolic blood pressure: 65-83 (females)/64-83 (males) [21].

Patients' vital signs	Upon admission	After supportive care
Heart rate (HR, beats/min)		
Case A (15 years)	100	89
Case B (13 years)	105	78
Blood pressure (BP, mmHg)		
Case A (15 years)	124/81	107/58
Case B (13 years)	134/73	102/52

**Table 2:** Toxicological analysis upon admission.

Test	Case A (15 years)	Case B (13 years)
Serum nicotine	134 ng/ml	266 ng/ml
Urine nicotine	2153 ng/ml	83 ng/ml
Screened substances	Negative for opioids, cannabinoids, benzodiazepines, amphetamines, coca alkaloids, and ethanol	Negative for opioids, cannabinoids, benzodiazepines, amphetamines, coca alkaloids, and ethanol

## Discussion

Although the literature on acute nicotine poisoning is extensive, recent cases related to the growing popularity of nicotine pouches highlight significant gaps in knowledge that require further investigation. This report of two incidents in minors contributes to the growing literature on the clinical manifestations of poisoning from these products and provides toxicological data to support the diagnosis.

Re-evaluating the lethal dose of nicotine

The traditionally accepted lethal dose of nicotine (LD 50) for an adult, ranging between 30 and 60 mg, is now strongly challenged. This value originates from a 1906 German textbook, "Kompendium der gerichtlichen Medizin" by pharmacologist Rudolf Kobert, which in turn was based on informal reports from 19th-century self-experiments [18,22]. The methodology of these early studies was highly problematic according to modern scientific standards. Observations were based on a minimal sample, the environment was uncontrolled, the purity of the nicotine was questionable, and there was no significant correlation between blood nicotine levels and patient outcomes. Nevertheless, this inaccurate estimate was established as "standard" in many toxicology and pharmacology textbooks for decades. The continued use of outdated and methodologically flawed scientific information is a notable phenomenon in the medical community. This analysis reveals how old data can persist for long periods, which can lead to a mistaken perception of risk, either overestimating toxicity at low doses or underestimating the possibility of survival at higher ones. Today, studies such as that by Bernd Mayer (2014) argue that the actual lethal dose for an adult is likely to range between 500 and 1000 mg (or 6.5-13 mg/kg), an estimate that aligns better with experimental data from animals. This re-evaluation emphasizes the need for modern, in-depth studies on nicotine toxicity, especially in the context of new products on the market [23].

Clinical-toxicological correlation and individual sensitivity

There is insufficient data to determine a specific threshold of blood nicotine level that causes symptoms of acute poisoning. The clinical response to nicotine is highly variable and depends on factors such as age, body weight [23-25], pre-existing nicotine tolerance (e.g., from prior smoking), and general health status. The two cases presented here are a characteristic example of this variability. The 13-year-old girl showed more severe symptoms (including loss of consciousness) in just 20 minutes, with a serum nicotine level of 266 ng/ml. In contrast, the 15-year-old patient, with milder symptoms, had a serum nicotine level of 134 ng/ml, but the onset of symptoms was delayed by 90 minutes. This apparent mismatch between nicotine levels and symptom severity can be attributed to hidden factors, such as nicotine tolerance. The 13-year-old patient reported a history of occasional nicotine pouch use, which suggests that her body, already accustomed to nicotine, may have absorbed a larger quantity more quickly, leading to the rapid onset of symptoms. Conversely, for the 15-year-old patient who did not report previous use, his body may have absorbed nicotine more slowly, with symptoms appearing with a delay. For clinicians, this suggests that a single measurement of blood nicotine levels upon admission is not always sufficient to determine the severity of poisoning. Instead, the clinical picture and use history are more reliable indicators for case management. For example, it has been reported that even a small acute intake of nicotine from pouches at a dose of just 0.8 µg/kg of body weight can cause an increase in heart rate [26].

Regulatory framework and public health implications

Nicotine pouches have emerged as a new category of products at the center of public health interest. While the international literature contains conflicting findings regarding their use, international health organizations, tobacco companies, and governments agree on two central points: that these products are addictive and that they are not intended for individuals under 18 years of age. As far as the situation in Greece is concerned, the recent Greek Law 5216/2025 establishes a coherent framework in which new tobacco products, e-cigarettes, refilled containers, and herbal products are fully equated with traditional cigarettes, and there are strict bans on the sale and distribution of tobacco and nicotine products to minors. More specifically, verification by sellers of the consumer’s age through physical or digital identification is now obligatory [27].

Nevertheless, consumption of nicotine pouches by adolescents and even children is constantly increasing, despite the fact that the FDA has classified them as modified risk tobacco products (MRTPs) and not as nicotine replacement therapies (NRTs) [28]. This distinction reflects a fundamental difference in their purpose and legal status. Specifically, while the use of NRTs is medically prescribed for smoking cessation, MRTPs are sold with the claim of reducing harm or the risk of tobacco-related diseases, compared to other available products. While the debate often focuses on their role in harm reduction for adults, the most serious public health concern revolves around their increasing use by young people. This increase is a result of targeted marketing strategies, similar to those historically used for cigarettes. Their promotion as "tobacco-free" products creates the mistaken perception that they are harmless. Furthermore, widespread promotion on social media has linked their use with images of confidence and masculinity, directly attracting the young audience through "Zynfluencers". Their discreet packaging, which can resemble candy tins, also makes them easy to hide from parents and educators. The absence of a unified regulatory framework, especially in the European Union, exacerbates the problem. It is of vital importance to strengthen awareness campaigns aimed at young people. These campaigns must focus on the inherent dangers of nicotine, a highly addictive substance, to the developing adolescent brain, regardless of the product that delivers it.

Recommendations from the literature

This report of two cases of acute nicotine poisoning in adolescents, following the use of nicotine pouches, underscores a real and existing public health risk. Although the patients responded positively to supportive care, these cases highlight the need for greater clinical vigilance. The analysis of the cases and the literature review lead to the following findings and recommendations.

Clinical Awareness

Emergency department physicians should include acute nicotine poisoning in the differential diagnosis of patients, regardless of age, who present with a compatible clinical picture (e.g., gastrointestinal, neurological, or cardiovascular symptoms), even if they do not report a history of using tobacco or other nicotine products.

Individual Variability 

The response to nicotine toxicity is highly individual and is affected by factors such as body weight and pre-existing tolerance. A single measurement of blood nicotine levels upon admission is not always sufficient to explain the severity of symptoms. More and more in-depth studies are needed to correlate the clinical picture with the pharmacokinetics of nicotine, especially in relation to its different modes of absorption, and to provide data on monitoring blood levels.

Re-evaluating Toxicology

The acceptance of outdated and methodologically questionable data, such as the historical value of the lethal dose of nicotine, needs to be re-evaluated. Extensive research is required to reassess the toxic and lethal dose, especially for minors.

Need for Legislative Regulation

The distinction between a "reduced harm" product for adult smokers and a "risk-free" product for minors is critical. Governments and health organizations must strengthen the legal framework to prevent the illegal sale and aggressive promotion of nicotine pouches to children and adolescents, as these products constitute a significant gateway to nicotine addiction for a new generation.

## Conclusions

The presented cases of acute nicotine poisoning in two adolescents following the use of nicotine pouches suggest that these products may pose an underrecognized clinical and public health risk to minors. Despite being marketed as “reduced-harm” alternatives for adult smokers, nicotine pouches are capable of delivering high and rapidly absorbed doses of nicotine through the oral mucosa, leading to significant toxic effects even after short-term use. The variability in symptom onset and severity observed between the two patients highlights the complex pharmacokinetics of nicotine and the influence of individual factors such as age, body weight, and prior nicotine exposure. These findings reinforce the need for clinicians to maintain a high index of suspicion for nicotine intoxication in adolescents presenting with compatible gastrointestinal, neurological, or cardiovascular symptoms, regardless of reported smoking history.

From a public health perspective, these cases highlight the urgent need to address the gap between the perception and the reality of nicotine pouch safety. The widespread availability, appealing flavors, and aggressive marketing of these products contribute to their increasing use among adolescents, despite legal restrictions. Strengthening regulatory frameworks, enforcing age-related sales bans, and implementing targeted educational campaigns are essential to prevent misinterpretation of “reduced harm” claims as “risk-free.” Ultimately, protecting minors from nicotine exposure requires coordinated action by healthcare professionals, educators, and public health authorities, alongside continued research into the toxicological profile of emerging nicotine delivery products.
